# Responses of beech and spruce foliage to elevated carbon dioxide, increased nitrogen deposition and soil type

**DOI:** 10.1093/aobpla/plv067

**Published:** 2015-06-19

**Authors:** Madeleine Silvia Günthardt-Goerg, Pierre Vollenweider

**Affiliations:** Forest Dynamics, Swiss Federal Institute for Forest, Snow and Landscape Research WSL, Zürcherstrasse 111, CH-8903 Birmensdorf, Switzerland

**Keywords:** Cell structure, chlorophyll, climate change, condensed tannins, elevated CO_2_, *Fagus sylvatica*, mineral nutrition, *Picea abies*

## Abstract

Although enhanced carbon fixation by forest trees may contribute significantly to mitigating an increase in atmospheric carbon dioxide (CO_2_), capacities for this vary greatly among different tree species and locations. This study compared reactions in the foliage of a deciduous and a coniferous tree species (important central European trees, beech and spruce) to an elevated supply of CO_2_ and evaluated the importance of the soil type and increased nitrogen deposition on foliar nutrient concentrations and cellular stress reactions. During a period of 4 years, beech (represented by trees from four different regions) and spruce saplings (eight regions), planted together on either acidic or calcareous forest soil in the experimental model ecosystem chambers, were exposed to single and combined treatments consisting of elevated carbon dioxide (+CO_2,_ 590 versus 374 μL L^−1^) and elevated wet nitrogen deposition (+ND, 50 versus 5 kg ha^−1^ a^−1^). Leaf size and foliage mass of spruce were increased by +CO_2_ on both soil types, but those of beech by +ND on the calcareous soil only. The magnitude of the effects varied among the tree origins in both species. Moreover, the concentration of secondary compounds (proanthocyanidins) and the leaf mass per area, as a consequence of cell wall thickening, were also increased and formed important carbon sinks within the foliage. Although the species elemental concentrations differed in their response to CO_2_ fertilization, the +CO_2_ treatment effect was weakened by an acceleration of cell senescence in both species, as shown by a decrease in photosynthetic pigment and nitrogen concentration, discolouration and stress symptoms at the cell level; the latter were stronger in beech than spruce. Hence, young trees belonging to a species with different ecological niches can show contrasting responses in their foliage size, but similar responses at the cell level, upon exposure to elevated levels of CO_2_. The soil type and its nutrient supply largely determined the fertilization gain, especially in the case of beech trees with a narrow ecological amplitude.

## Abstract (precis)

Over four years young central European beech and spruce trees, growing on either acidic or calcareous forest soil, were exposed to elevated CO_2_ and nitrogen deposition as forecast for the period 2050–2100. The elevated CO_2_ had a positive fertilising effect on spruce foliage only, but led to an accumulation of tannins, cell wall thickening and an acceleration of cell senescence in both species. These effects were partly mediated by the soil type and nitrogen supply. Rising CO_2_ concentration and nitrogen deposition may have similar effects on the leaf cell physiology - mechanistically - but induce contrasting, specific growth responses.

## Introduction

During the 20th century and with anthropogenic activities being the primary cause, emissions of carbon dioxide (CO_2_) and the nitrogen deposition (ND) have increased sizeably with no levelling off of CO_2_ in sight for the second decade of the 21st century. To halt the forecasted climate warming by 2050, abatement of present greenhouse gas emissions by 50 % would be needed but is impeded by different factors including timescale constraints and social acceptance (Hansen *et al*. 2013).

As a greenhouse gas, CO_2_ is the main contributor to the ongoing global warming whilst being, together with mineral nutrients (e.g. nitrogen (N)), water, sunlight and appropriate temperatures, one of the main prerequisites for plant growth. Accordingly, elevated CO_2_ concentrations can act as a fertilizer and promote productivity, as observed in the case of poplar trees treated over 6 years in the POP/EUROFACE experiment (Liberloo *et al*. 2009) or those exposed to elevated CO_2_ during 11 years in the Aspen FACE experiment at Rhinelander, Wisconsin, USA (Talhelm *et al*. 2014). However, coppicing and an unlimited supply of light, water and nutrients may also have contributed to these findings, whereas this fertilizing effect progressively vanished during the first 7 years of exposure (Kubiske *et al*. 2006). Reviewing the results from several FACE experiments, Norby and Zak (2011) concluded that the enhanced growth rates observed in trees in response to elevated CO_2_ can level off over time and that transient changes in assimilated carbon pools need further research in the future. As a consequence of increased competition and/or larger water and nutrient requirements, older rather than younger trees, especially within undisturbed forest stands, appear less responsive to an enhanced carbon supply (McCarthy *et al*. 2010; Ryan 2013). Hence, in the Swiss Canopy Crane experiment, no significant biomass increase of the 100-year-old trees belonging to five deciduous tree species was measured after 8 years of exposure to elevated CO_2_ (Bader *et al*. 2013). Interestingly, photosynthesis responded positively to treatment, but the fate of supplementary assimilates remained unresolved. The response of trees to elevated CO_2_ can be further complicated by synergistic or antagonistic interactions with environmental constraints such as drought (van der Molen *et al*. 2011), pests, infections, pollutants, nutrients and other soil properties (Karnosky 2003; Zak *et al*. 2003). The lower N concentration measured in the foliage of young trees from various species exposed to elevated CO_2_ was interpreted as a dilution effect resulting from an enhanced carbon assimilation (Hättenschwiler and Körner 1998; Eller *et al*. 2011) and relievable by N fertilization (Esmeijer-Liu *et al*. 2009). In studies with herbaceous plants, the lower leaf N content related to a decreased N uptake which contributed to a depression of the initially enhanced CO_2_ assimilation eventually leading to accelerated leaf senescence (Makino and Mae 1999). Indeed, reduced leaf N content and decreased photosynthesis are classical drivers and markers of accelerated cell senescence (ACS, Pell *et al*. 1999). Consequences of the autumnal senescence later in the vegetation season are unclear with studies, using various tree species and experimental settings, showing leaf fall acceleration (Warren *et al*. 2011), retardation (Taylor *et al*. 2008; Vapaavuori *et al*. 2009) or no effect on foliage shedding (Herrick and Thomas 2003; Asshoff *et al*. 2006). The varying sensitivity of species or the missing specificity of leaf discolouration symptoms with regard to environmental constraints (Vollenweider and Günthardt-Goerg 2006) may contribute to inconsistencies between studies whereas the assessment of reactions at cell level can ascertain a more accurate diagnosis by more precisely identifying the involved stress factor (e.g. biotic infection, nutrient deficiency, oxidative stress, drought) (Fink 1999; Günthardt and Vollenweider 2007). However, the influence of elevated CO_2_ and N deposition on a possible ACS, in deciduous versus evergreen foliage, has not been studied so far.

Higher amounts of fixed carbon can be allocated to various sinks. In plants, by growing in CO_2_-enriched air, the carbon surplus can be invested into (i) non-structural carbohydrates, including starch (Kainulainen *et al*. 1998; Oksanen *et al*. 2005), (ii) polysaccharides and (iii) secondary compounds, often phenolics. Increased production of polysaccharides can contribute to cell wall thickening and higher specific leaf mass per area (LMA), as observed in foliage of various conifer and broadleaved tree species in response to elevated CO_2_ (Tognetti and Johnson 1999; Oksanen *et al*. 2005; Eller *et al*. 2011; Pokorny *et al*. 2011). Regarding secondary compounds, the foliage response to elevated CO_2_, as measured in different conifer and broadleaved species, is still unclear with either unchanged (Kainulainen *et al*. 1998; Räisänen *et al*. 2008), increased (Tognetti and Johnson 1999; Sallas *et al*. 2001; Veteli *et al*. 2007; Vapaavuori *et al*. 2009) or decreased (Peñuelas *et al*. 1996) amounts of phenolic compounds. According to the growth–differentiation balance hypothesis (GDBH)—which states a trade-off in plant internal resource allocation between growth and differentiation processes, including defense (Bezemer *et al*. 2000; Matyssek *et al*. 2012)—an increased assimilate partitioning in favour of phenolic compounds is expected in response to exposure to elevated CO_2_ (Mattson *et al*. 2005). Higher concentrations of phenolic compounds can also denote stress reactions to various environmental constraints and are observed in the case of degenerative processes leading to ACS (Günthardt-Goerg and Vollenweider 2007). Interestingly, shrubs growing in a natural CO_2_ spring—and thus adapted to long-term elevated CO_2_—did not show any change in non-structural carbohydrates and secondary compounds (Peñuelas *et al*. 2002). This suggests that many experimental findings may relate primarily to transient changes.

The main objectives in this study were to compare foliage reaction to elevated CO_2_ and N deposition of the two important central European species (*Fagus sylvatica* L. and *Picea abies* (L.) H. Karst) as a function of the soil nutrient availability. With a view to mechanistic understanding of reactions in two species, with contrasted ecological niche (Härdtle *et al*. 2004) and with deciduous versus evergreen foliage, we tested the following hypotheses in a 4-year study: (i) elevated CO_2_ differently affects the leaf versus needle morphology, primary and secondary metabolism and element content during the vegetation season as a function of the species, nitrogen supply and soil type; (ii) the enhancement of a CO_2_ supply causes changes in nutrient demand which can be remediated by elevated N deposition and (iii) a decreased CO_2_ fertilization effect is associated with degenerative structural changes within leaves and needles indicative of ACS. Therefore, and in the framework of the 4-year ICAT (Impact of elevated CO_2_ levels and Air pollution on Tree physiology) experiment (Egli *et al*. 1998; Spinnler *et al*. 2003), we focused on responses in deciduous tree leaves versus long-living evergreen needles at cell to organ level, as indicated by changes in the leaf morphology, biochemical indicators of primary and secondary metabolism, element content and tissue and cell structure.

## Methods

### Experimental design

The experiment was carried out in the model ecosystem facility (MODOEK, http://www.wsl.ch/fe/walddynamik/projekte/modoek/index_EN) of the Swiss Federal Research Institute for Forest, Snow and Landscape Research WSL at Birmensdorf, Switzerland (8°27′23″E, 47°21′48″N, 545 m above sea level) from May 1995 to October 1998. The MODOEK consists of 16 large glass-walled and hexagonal open-top chambers (height 3 m, area 6.7 m^2^, aboveground volume 20.1 m^3^) arranged in a Latin square design with four treatments replicated four times each:

- Control: ambient air and ambient ND (supplied in the form of NH_4_NO_3_ by irrigation)
- +CO_2_: elevated CO_2_, with the addition of 200 μL L^−1^ CO_2_ during the growing season (May–October) to ambient air, as a moderate value among the forecasted categories for the year 2050 (445–1130 μL L^−1^, IPCC 2007); ambient N deposition
- +ND: elevated N deposition with 10-fold enhancement of NH_4_NO_3_ concentrations, as forecasted for the period 2050–2100 (IPCC 2007); ambient air
- +CO_2_ + ND: elevated CO_2_ and ND.

From 07.00 to 19.00 h, the mean daily CO_2_ concentrations (±standard deviation) over the 4-year duration of experiment in the control/+CO_2_ treatment amounted to 372 ± 16/581 ± 87 μL L^−1^, and from 19.00 to 07.00 h to 413 ± 38/603± 73 μL L^−1^. The seasonal ND (kg ha^−1^) in the control/+ND treatment amounted to 2.6/25.7 during the first, 6.1/61 during the second, 7.1/71.3 during the third and 7.4/74.3 during the fourth experimental season.

From May to October, the transparent roofs of MODOEK automatically closed at the onset of rain but were kept open to allow natural precipitation, including snow, during wintertime. Throughout the growing season, plants were irrigated during the night to field capacity (monitored using the soil water content) by means of 12 sprinklers per chamber, mounted above the canopy. These sprinklers provided synthetic rain, consisting of water purified by electro-osmosis with an ionic composition equivalent to the last 30 years' mean natural precipitation at the experimental site (pH 5–6; 0.2 Ca, 0.6 Cl, 0.3 K, 0.03 Mg, 0.1 Na, 0.1 P, 0.3 SO_4_, 0.01 Zn mg L^−1^). Given the plants' requirements, 360 L m^−2^ water were supplied in the first, 694 in the second, 848 in the third and 864 in the fourth year of experiment.

Each MODOEK chamber was split belowground into two 1.5-m deep concrete-walled lysimeter compartments with a surface area of 3 m^2^. Each lysimeter was filled with pure quartz gravel (30 cm), quartz sand (20 cm) and 60 cm of forest sub-soil and 40 cm of topsoil either ‘acidic’ from a sandy loam brown soil (Haplic Alisol, pH 3.8 in 0.01 M CaCl_2_) or ‘calcareous’ from an alluvial calcareous loamy sand soil (Calcareous Fluvisol, pH 7.0 in 0.01 M CaCl_2_). These two soils originated from natural spruce/beech forest stands in the Aare (calcareous) or Rhine (acidic) valley, Switzerland and were randomly attributed to either the north or south compartment of each chamber. The topsoil properties were acidic/calcareous: Ca 14.3/124 and K 1.2/0.6 meq, P 2.1/6.5 and N (exchangeable KCl) 2.4/3.8 mg kg soil^−1^ (detailed soil analyses in Sonnleitner *et al*. 2001).

Per lysimeter, eight European beech (*Fagus sylvatica*) and eight Norway spruce (*Picea abies*) saplings 30–40 cm high and with roots trimmed to 10 cm prior to planting were planted during the autumn (October) preceding the first experimental year at positions fixed for species but randomized for tree origins (16 trees per soil compartment and 512 trees in total). Different plant material (according to the seedling availability) was used to ascertain the species reaction irrespective of the seedling propagation, age or genetic constitution. The tree origin refers to the geographical location of the population where the seeds were originally harvested to generate the trees used in the experiment. Trees were either grown directly from seed (beech, spruce origins 7 and 8) or from clonal cuttings (spruce origins 1–6) rooted prior to starting the experiment. Beech seeds originated from four Swiss midland populations (Aar, Aarburg; Hir, Hirschtal; Her, Herzogenbuchsee; Sih, Sihlwald) and were 2- (Aar, Hir) or 3-years (Her, Sih) old by the time of planting (two seedlings per origin in each soil compartment). Spruce origins included three German (1 Harzvorland, 2 Hochsauerland, 3 Frankenwald), one Romanian (4 Carpathia) and four Swiss midland populations (5 Kerns, 6 Neuwilen, 7 Bremgarten, 8 Maschwanden) either 2- (1–4) or 4-years old (5–8).

### Sampling and measurement of tree growth and foliage reactions

During the 4 years of experimentation, the foliage of beech and spruce saplings was sampled twice a year in 20–22 July and 16–18 September (before autumnal discolouration). For beech, foliage aliquots, consisting of four representative and healthy leaves, were sampled throughout the tree crown whereas for spruce, aliquots of 20 needles were excised from the middle of current-year lateral and previous-year twigs after selecting one branch from the second highest whirl. To determine the leaf water content, the foliage samples were weighed upon sampling and after oven-drying at 65 °C. At the end of the experiment, trees were harvested and the total foliage mass of current- (beech and spruce), previous-year and older foliage fractions (spruce) was determined.

On each aforementioned fresh foliage, aliquot changes in the leaf morphology were characterized by scanning the leaf area (Delta-T area meter MK2) and measuring the needle length. To estimate the total surface area and mean thickness of each needle generation, the cross-sectional area, diameter and perimeter of cross sections trimmed from four fresh needles per sample were determined by light microscopy and image analysis (Leica Quantimet 500+ system, Leica, Cambridge, UK).

Changes in the leaf and needle colour were evaluated using colour charts (Biesalski 1957) and converting readings into a semi-quantitative rank variable with a scale from 0 (yellow or brown) to 10 (dark green). Biochemical markers of primary and secondary metabolism consisted of the light-adapted (midday) photosynthetic pigments of chloroplasts and of the most abundant phenolic—i.e. proanthocyanidin (PC = condensed tannins)—fraction. Pigment analyses were carried out as reported in Wonisch *et al*. (2001) using foliage samples from two origins per species (seedlings of different age) harvested in July during the last experimental year. For leaf photosynthetic pigment (chlorophyll *a* and *b*, α- and β-carotenoids) analysis, foliage was immediately frozen in liquid nitrogen. Plant powder (100 mg) was extracted two times in acetone (1 mL, 1 min on a Vortex-mixer) and, after centrifugation at 4 °C, the supernatants were combined and adjusted to a final volume of 3 mL. These acetone extracts were injected (20 µL) using a cooled (0 °C) auto-sampler. Analyses were carried out by an HPLC gradient method: Column Spherisorb S5 ODS2 250 × 4.6 mm with precolumn S5 ODS2 50 × 4.6 mm; solvent A: acetonitrile : methanol : water = 100 : 10 : 5 (v/v/v); solvent B: acetone : ethyl acetate = 2 : 1 (v/v); linear gradient from 10 % solvent B to 70 % solvent B in 18 min; run time 30 min; flow rate 1 mL min^−1^; and photometric detection 440 nm. The PC concentration was measured using 1 g of shock-frozen and freeze-dried leaf/needle material sampled in one origin per species (beech: Hir; spruce: number 8, Maschwanden) in July and September of the last experimental year. For PC extraction, the material was frozen in liquid nitrogen prior to 1 min homogenization in a B. Braun Mikro-dismembrator II (60 × 15 mm oscillations s^−1^) using stainless steel balls. The still frozen powder was transferred to a separating funnel and extracted four times during 3 min under magnetic stirring at room temperature with 4.5 mL acetone 70 % containing 0.1 % ascorbic acid. The clear filtrates were combined and partially purified according to Broadhurst and Jones (1978). Proanthocyanidins were quantified using the acid-vanillin (mainly the oligomers, OPC, Broadhurst and Jones 1978; Waterman and Mole 1994) and PC (mainly the polymers, PPC, Porter *et al*. 1986; Waterman and Mole 1994) assay. This latter assay was also used to quantify PC in the insoluble and primarily cell wall fraction. Absorbance was read on a UV-160 spectrophotometer (Shimadzu, Kyoto, Japan) and results are expressed as (+)catechin (acid-vanillin assay) and perlargonidin (PC assay) equivalents.

The concentration and ratio of leaf elements (C, N, C/N, Ca, Fe, K, Mg, Mn, P, P/N, S, Zn) were determined using foliage samples from two to four (last experimental year) origins per species harvested in July and September. Samples were milled, dissolved using high-pressure digestion (240 °C; 120 bar) and analysed in duplicates (spread <10 %) in the central laboratory of WSL using a gas chromatograph (NC-2500, Carlo Erba-Instruments, Wigan, UK) for C and N and by ICP-OES (Optima 7300DV by Perkin Elmer Inc., MA, USA) for the other elements.

Microscopic analyses of the structural changes at cell level in response to treatments were carried out using foliage samples excised in July, September and January (spruce only) of each experimental year, selecting the same two origins per species as for elemental and pigment analyses. Samples were used fresh or fixed and examined by light microscopy (LM), fluorescence and transmission electron microscopy (TEM). Fresh samples were cut with a hand microtome to 50 µm, embedded samples with a Reichert Ultramicrotome to semi-thin 2 µm and ultra-thin 90 nm TEM sections. Sections were stained using different metachromatic or specific histochemical stains (toluidine blue, vanillin, *p*-dimethylaminocinnamaldehyde (DMACA) for LM; coriphosphine for fluorescence) or contrasted (TEM). Detailed methods are given by Günthardt-Goerg *et al*. (1997) and Vollenweider *et al*. (2003). Structural changes by the treatments within each origin, at each harvesting date and on both soils were compared pairwise with the corresponding control and documented with micrographs. Changes between the acidic to the calcareous soil within each treatment, origin and date were assessed in a similar way. Severity changes—based on several micromorphological traits each—were evaluated using rank estimates with four levels (unchanged/low/medium/severe). A change was considered to be effective when consistent reactions over the whole experimental period were observed.

### Statistical analysis

The main significant differences between species, treatments, soils, origins of the plant material and harvest times in the season and their interactions were tested using variance analysis (ANOVA/GLM procedures, SAS Institute, Inc., Cary NC, USA, version 9.1). The statistical unit was the tree with generally one tree replicate per origin and soil compartment. For beech, in the case of biomass, leaf colour and size measurements, individual values represented averages of two trees per origin and soil compartment. Species were analysed separately. The mean values per species from different origins were used to calculate the species difference and their interactions with the treatments, soils and harvest dates. Whatever the sampling date, data from the first experimental year showed significant differences with those from subsequent years whereas results during later years were similar. This first year effect at the beginning of the experiment was attributed to the still ongoing acclimation processes to MODOEK conditions and consequently, this first year of data was discarded whereas the values from subsequent years were pooled together and the factor year not further considered. All data distributions were successfully tested for normality (Shapiro). In addition, statistical tests (*F*-tests) were performed on the levels given by the hierarchical structure of the experimental layout with post-hoc pairwise Tukey's studentized range (HSD) test. For the ranked variable (leaf colour), differences between groups were confirmed in all cases using non-parametric testing (SAS npar1way). However, because the results were similar to those using ANOVA, we decided, for consistency, to present the same calculations for all parameters.

## Results

### Responses in European beech

**Figure 1.**
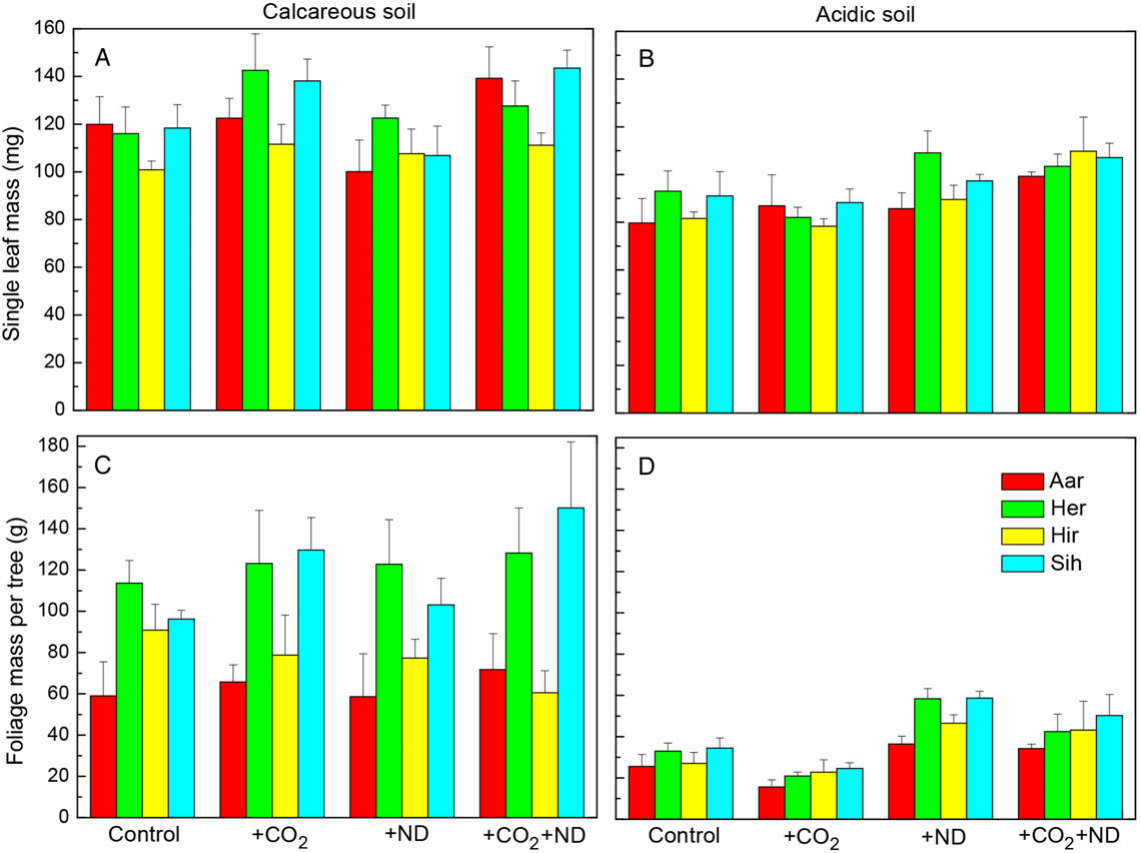
The change in the dry mass of single leaves (A and B) and total crown foliage (C and D) in beech in response to +CO_2_, +ND and +CO_2_ + ND versus control for several origins (Aar, Aarburg; Her, Herzogenbuchsee; Hir, Hirschtal; Sih, Sihlwald) growing together on either acidic or calcareous forest soil (mean values + SE, *N* = 4, September harvest).

The treatments changed the morphology of beech leaves and their effect varied as a function of the soil type and plant origin. Over the three vegetation periods, the dry leaf mass (Fig. 1A and B), area and thickness of single leaves were increased by 12, 7 and 6 % on average in response to +CO_2_ on the calcareous soil, whereas on the acidic soil they were significantly increased by +ND (11, 8 and 6 %, significant treatment × soil interaction, Table 1). Total leaf mass was only increased by ND by 30 % on acidic soil, but unchanged by +CO_2_. The soil type had a strong influence on the leaf-level response to +CO_2_ and the leaf mass, area, thickness and LMA were by 17, 10, 7 and 9 % lower, respectively, on the acidic versus calcareous soil, whole-tree foliage mass even by 62 % (Fig. 1C and D, Table 1). With significant differences between origins, the leaf water content was on average 4 % lower in September than in July (whilst the LMA showed no change) but was not responsive to the treatments. In contrast to the dry leaf mass and area (<8 % difference among the origins) and related to initial seedling age, the total foliage dry mass by the end of experiment showed a doubled biomass of Her and Sih compared with Aar and Hir on the nutrient-rich calcareous soil (Fig. 1C, Table 1).

**Table 1.** Analysis of variance, significance levels of factors affecting beech foliage parameters. Effects (*F*-values above with df as subscripts, *P*-values, significant in bold, in parentheses, ↑ increasing, ↓ decreasing) of treatments (+CO_2_, +ND), season (harvest month September versus July), plant origin and soil type (acidic versus calcareous), number of repetitions each = 4, on the crown foliage per tree or single leaf biomass, morphology, biochemistry and leaf elemental concentrations. LMA, leaf dry mass per area; OPC, oligo-proanthocyanidins; PPC, polymerized proanthocyanidins. The interaction CO_2_ × ND was not significant in any case.

		Season	Origin	+CO_2_	+ND	Soil	Treatment × soil interactions
Tree	Foliage	–	12.1_3,71_ (**<0.001**)	0.3_1,71_ 0.603	23.5_1,71_ (**0.003**)↑	294.1_1,71_ (**<0.001**)↓	CO_2_ × soil 7.5_1,71_ (**0.017**), ND × soil 7.3_1,71_ (**0.018**)
Single leaf	Mass	1.7_1,167_ 0.191	2.7_3,167_ 0.046	24.5_1,167_ (**0.003**)↑	12.1_1,167_ (**0.013**)↑	108.0_1,167_ (**<0.001**)↓	CO_2_ × soil 8.6_1,167_ (**0.012**), ND × soil 10.0_1,167_ (**0.008**)
	Area	1.1_1,167_ 0.301	1.1_3,167_ 0.334	7.6_1,167_ (**0.033**)↑	5.2_1,167_ 0.063↑	34.5_1,167_ (**<0.001**)↓	ND × soil 6.1_1,167_ (**0.028**)
	Thickness	0.46_1,167_ 0.501	3.8_,167_ (**0.011**)	3.6_1,167_ 0.107	1.2_1,167_ 0.31	56.0_1,167_ (**<0.001**)↓	CO_2_ × soil 6.8_1,167_ (**0.022**)
	% water	61.9_1,167_ (**<0.001**)↓	4.3_3,167_ (**0.006**)	0.5_1,167_ 0.496	1.1_1,167_ 0.333	2.5_1,167_ 0.139	
	LMA	2.4_1,167_ 0.126	13.0_3,167_ (**<0.001**)	33.4_1,167_ (**0.001**)↑	19.5_1,167_ (**0.005**)↑	40.7_1,167_ (**<0.001**)↓	
	Colour	10.6_1,167_ (**0.001**)↓	2.1_3,167_ 0.109	104.2_1,167_ (**<0.001**)↓	44.9_1,167_ (**<0.001**)↑	14.3_1,167_ (**0.002**)↓	
	Chlorophyll *a* + *b*	–	12.0_1,48_ (**0.001**)	8.9_1,48_ (**0.004**)↓	1.4_1,48_ 0.250	0.0_1,48_ 0.921	
	α + β carotenoids	–	2.5_1,48_ 0.121	10.3_1,48_ (**0.002**)↓	0.9_1,48_ 0.357	3.2_1,48_ 0.079	
	OPC	0.4_1,30_ 0.523	–	6.6_1,30_ (**0.016**)↑	12.0_1,30_ (**0.002**)↑	0.5_1,30_ 0.484	
	PPC	14.3_1,30_ (**<0.001**)↓	–	4.9_1,30_ (**0.035**)↑	8.1_1,30_ (**0.008**)↑	0.9_1,30_ 0.522	
	PPC cell wall	17.7_1,30_ (**<0.001**)↑	–	0.1_1,30_ 0.836	0.6_1,30_ 0.434	0.1_1,30_ 0.775	
Element	C	0.0_1,117_ 0.906↑	52.7_3,117_ (**<0.001**)	0.0_1,117_ 0.969	2.1_1,117_ 0.195	18.6_1,117_ (**<0.001**)↓	
	N	68.2_1,117_ (**<0.001**)↓	21.0_3,117_ (**<0.001**)	36.2_1,117_ (**<0.001**)↓	15.9_1,117_ (**0.007**)↑	110.5_1,117_ (**<0.001**)↓	ND × soil 7.1_1,117_ (**0.020**)
	C/N	21.9_1,117_ (**<0.001**)↑	6.0_3,117_ (**0.001**)	26.2_1,117_ (**0.002**)↑	12.6_1,117_ (**0.012**)↓	91.0_1,117_ (**<0.001**)↑	ND × soil 9.0_1,117_ (**0.010**)
	Ca	38.2_1,117_ (**<0.001**)↑	7.5_3,117_ (**<0.001**)	0.1_1,117_ 0.730	0.8_1,117_ 0.412	335.1_1,117_ (**<0.001**)↓	
	Fe	56.2_1,117_ (**<0.001**)↑	0.4_3,117_ 0.727	2.1_1,117_ 0.194	0.1_1,117_ 0.766	17.0_1,117_ (**0.001**)↑	
	K	130.2_1,117_ (**<0.001**)↑	15.5_1,117_ (**<0.001**)	0.6_1,117_ 0.471	1.6_1,117_ 0.250	16.3_1,117_ (**0.001**)↑	
	Mg	39.1_1,117_ (**<0.001**)↓	3.3_3,117_ (**0.022**)	7.9_1,117_ (**0.031**)↓	1.9_1,117_ 0.221	172.6_1,117_ (**<0.001**)↓	
	Mn	24.0_1,117_ (**<0.001**)↑	7.3_3,117_ (**<0.001**)	0.5_1,117_ 0.519	9.0_1,117_ (**0.024**)↓	1605.3_1,117_ (**<0.001**)↑	ND × soil 8.7_1,117_ (**0.011**)
	P	41.9_1,117_ (**<0.001**)↑	3.3_3,117_ (**0.022**)	0.8_1,117_ 0.231	25.7_1,117_ (**0.002**)↓	2.7_1,117_ 0.125	CO_2_ × soil 9.9_1,117_ (**0.008**)
	P/N	117.6_1,117_ (**<0.001**)↑	9.5_3,117_ (**<0.001**)	22.8_1,117_ (**0.003**)↑	55.7_1,117_ (**<0.001**)↓	15.8_1,117_ (**0.002**)↑	CO_2_ × soil 13.1_1,117_ (**0.003**), ND × soil 14.8_1,117_ (**0.002**)
	S	0.1_1,117_ 0.809	8.7_3,117_ (**<0.001**)	54.1_1,117_ (**<0.001**)↓	6.4_1,117_ (**0.044**)↓	157.2_1,117_ (**<0.001**)↓	ND × soil 9.2_1,117_ (**0.010**)
	Zn	8.1_1,117_ (**0.005**)↑	0.7_3,117_ 0.542	9.4_1,117_ (**0.022**)↑	0.4_1,117_ 0.541	3.9_1,117_ 0.069↑	ND × soil 6.1_1,117_ (**0.028**)

**Figure 2.**
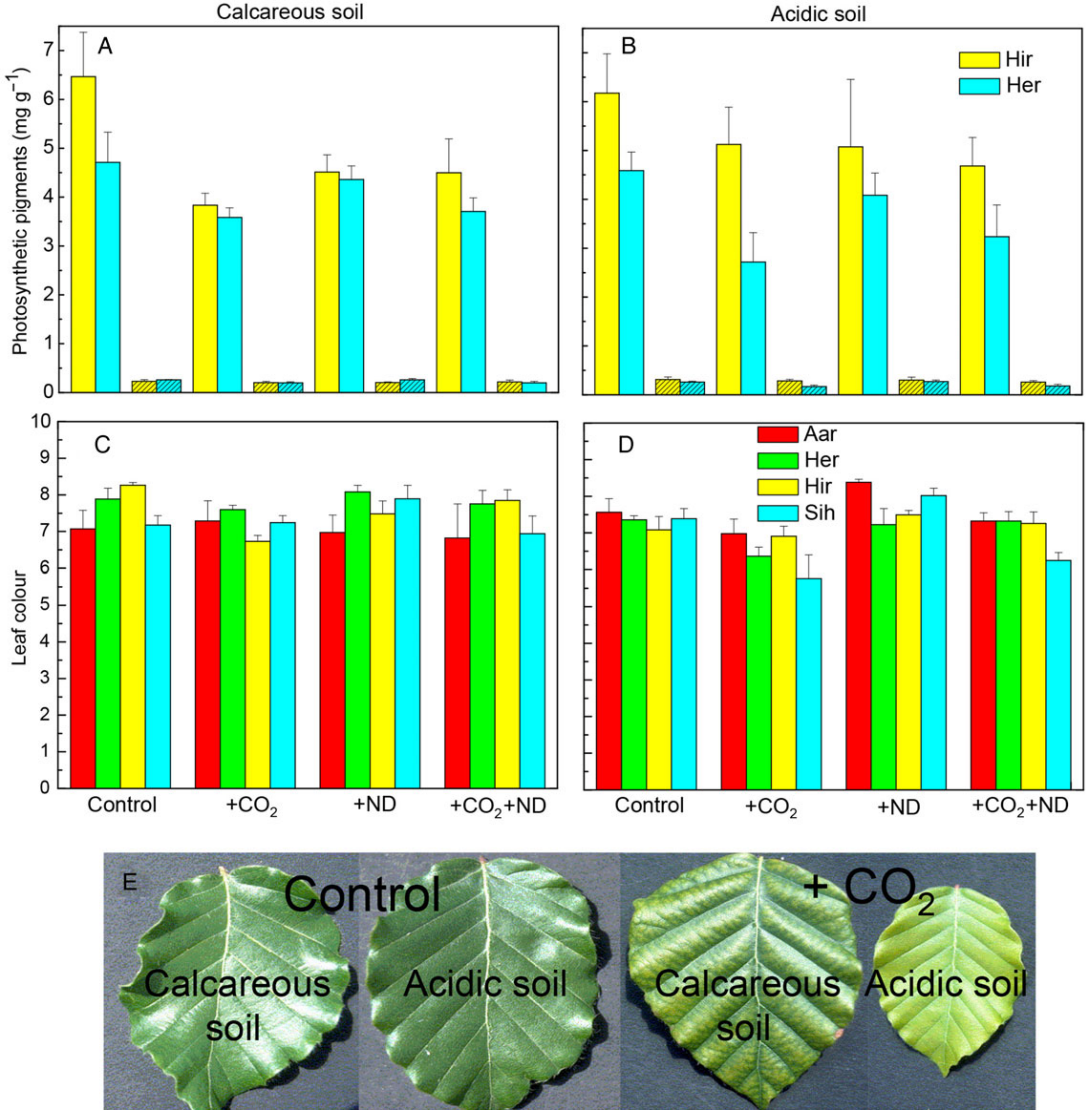
The change in the concentration of photosynthetic pigments in July (A and B), namely chlorophyll *a* + *b* and α + β carotenoids (hatched columns), and in the leaf colour in September (C and D) in response to +CO_2_, +ND and +CO_2_ + ND versus control, within the foliage of several origins of beech growing on either acidic or calcareous forest soil (mean values + SE, *N* = 4). Photographs (E) show typical examples of leaf discolouration in response to elevated CO_2_.

**Figure 3.**
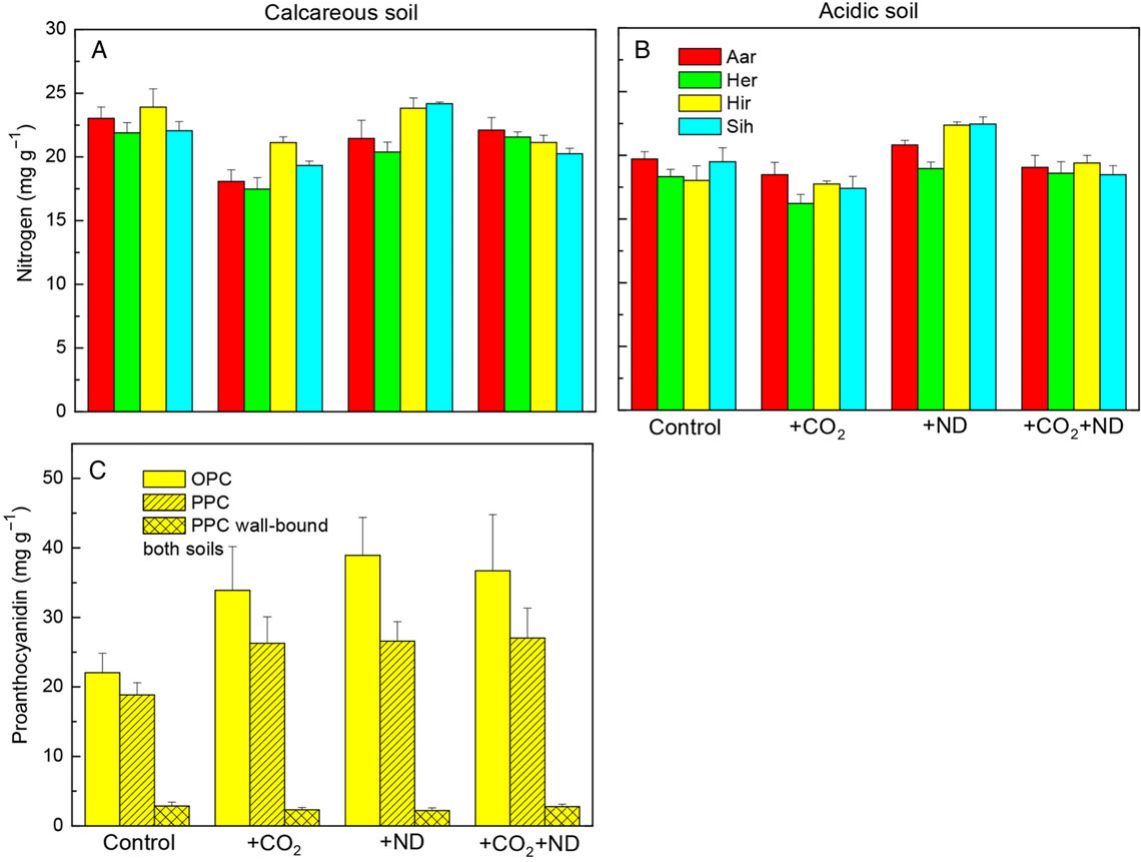
The change in the concentration of nitrogen (A and B) and PC (C) oligomers (OPC) and polymers (PPC) in September in response to +CO_2_, +ND and +CO_2_ + ND versus control, within the foliage of several origins of beech (C: Hir only) growing on either the acidic or calcareous forest soil (C: soil types without effect, data pooled); mean values + SE, A and B: *N* = 4, C: *N* = 8).

Exposure to +CO_2_ affected the photosynthetic pigment content and leaf colour of beech leaves, and this latter parameter varied as a function of the nitrogen supply and soil type. The leaf chlorophyll and carotenoid concentration on both soils was decreased in July by +CO_2_ by 30 and 20 %, respectively, whereas +ND caused no significant change (Fig. 2A and B, Table 1). In September, the colour of beech foliage showed over the experimental years lighter green hues in the +CO_2_ treatment (−5 % on calcareous, −11 % on acidic soil, Fig. 2C–E) whereas +ND led to darker green hue on acidic soil (+6 % Fig. 2D, Table 1). Accordingly, the N concentration in leaves was on average decreased by +CO_2_ (−11 %) but enhanced by +ND (+8 %; Fig. 3A and B, Table 1). Beech trees growing on the acidic versus calcareous soil also displayed an overall lighter green colour (−6 %, Fig. 2D versus C). An effect by the plant origin in the leaf chlorophyll content in July was transient, the differences levelling off by September.

Besides changes in the N content, the type of treatment and soil also affected other leaf elements, which remained within the normal range reported by Mellert and Göttlein (2012). Whilst showing only a small reaction to +CO_2_, the leaf level of phosphorus (P) was decreased by 14 % in response to +ND. Cross-changes of P and N resulted in a 33 % increase of the P/N ratio by +CO_2_ and a decrease by 21 % by +ND (Table 1). The S and Mg concentrations were correlated to the concentration of N (and also decreased by +CO_2_) whilst other elements showed only minor changes in response to the treatments. In general, leaf elements showed small but significant differences between the two soil types, in line with contrasting soil pH, whereas the foliar manganese (Mn) was 125 times higher, and calcium (Ca) and magnesium (Mg) 45 and 43 % lower, respectively, on the acidic soil. Seasonal changes were also observed with a decrease in foliar concentration of N and Mg by 8 and 17 %, respectively, and an increase in Ca/Fe/K/Mn/P/Zn between the July and September assessment amounting to 14/30/29/30/14/12 %, respectively. The variation between tree origins remained small.

**Figure 4.**
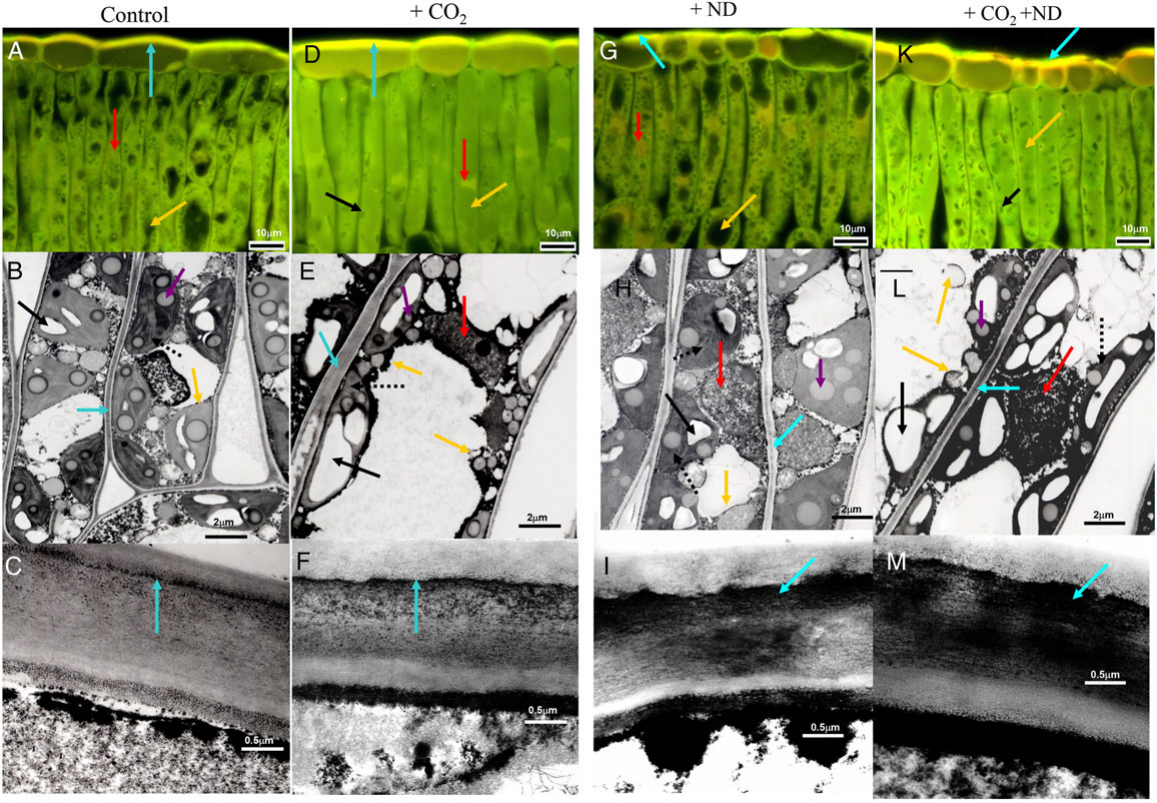
Structural effects of +CO_2_ (D–F), +ND (G–I) and +CO_2_ + ND (K–M) versus control (A–C) in the leaf blade cells of beech. Under +CO_2_, cells were enlarged (E versus B), cell walls thickened—primarily by pectin inlays within the outer wall layers (cyan arrows in D–F versus A, C), nuclear chromatin condensed (red arrows in D, E versus A, H) and vacuoles enlarged (yellow arrows in D, E versus A, B). The latter organelles also showed an irregular contour, as a consequence of plastoglobule extrusion and cytoplasm invagination, and they contained more phenolics. Moreover, chloroplasts showed characteristic changes including a lower frequency, a fuzzy grana and thylacoid structure and larger starch grains (dashed and black arrows in E versus B). Under +ND (G–I), a mostly intact cell structure was observed. As a tendency, electron-translucent plastoglobuli were enlarged (violet arrow H versus B), nuclei (red arrow) somewhat condensed and the size and frequency of starch grains (black arrow) and PC droplets slightly increased. The epidermal cell walls showed irregular thickening (G, I versus A, C). The +CO_2_ + ND treatment (K–M) triggered changes similar to those observed in response to +CO_2_. Staining with Coriphosphine for pectins (bright yellow), viewed at 450–490 nm excitation (A, D, G and K), TEM of palisade cells (B, E, H and L) and upper epidermal outer cell wall (C, F, I and M).

The foliar content of PCs and the cell structures primarily responded to the treatments. Independent of soil type, the concentration of soluble oligomeric OPC and polymerized PPC (vacuolar PC) in the +CO_2_ (+31 and +14 %, respectively) and +ND (+44 and +18 %, respectively) versus control treatment was markedly increased (Table 1, Fig. 3C), whereas the insoluble PPC (cell wall-bound PPC, Fig. 3C), amounts of which in September reached 10.2 % of those of soluble PPC (in July only 5.3 %, Table 1), showed no change. Microscopic changes were detected in July similar to those in mid-September both in the upper epidermis and in the upper mesophyll. In the upper epidermis, in comparison to control samples (Fig. 4A and C), cell walls were thickened in response to both +CO_2_ and +ND, primarily by pectin inlays within the outer wall layers (Fig. 4D, F, G and I) but in a more prominent and homogeneous way in the case of higher carbon availability (Fig. 4D, F, K and M). Cell walls in upper mesophyll of samples exposed to +CO_2_ were similarly thickened (Fig. 4E versus B). In response to the latter treatment, mesophyll cells showed structural changes indicative of degenerative processes, including the condensation of cytoplasm and nucleus and the enlargement of vacuoles (Fig. 4D, E versus A, B). Latter organelles were filled with condensed tannins and had an irregular periphery because of the extrusion of plastoglobuli. Further observations were a reduction in the number of chloroplasts, within chloroplasts grana and thylacoid structures were no longer clearly defined, there was an accumulation of large starch grains and the density of electron-translucent plastoglobuli was increased (Fig. 4E versus B). Mesophyll cells showed little changes by the +ND treatment. In comparison to control samples however, this treatment caused some enlargement of electron-translucent plastoglobuli and, similar to +CO_2_, tended to enhance the condensation of nucleus and increase the size and frequency of starch grains or PC droplets, but to a lesser extent (Fig. 4G, H versus A, B). Samples from the combined +CO_2_ + ND treatment (Fig. 4K–M) showed changes similar to those observed in response to +CO_2_. The tree origin or soil type did not influence these results.

### Responses of Norway spruce and comparison with beech

**Figure 5.**
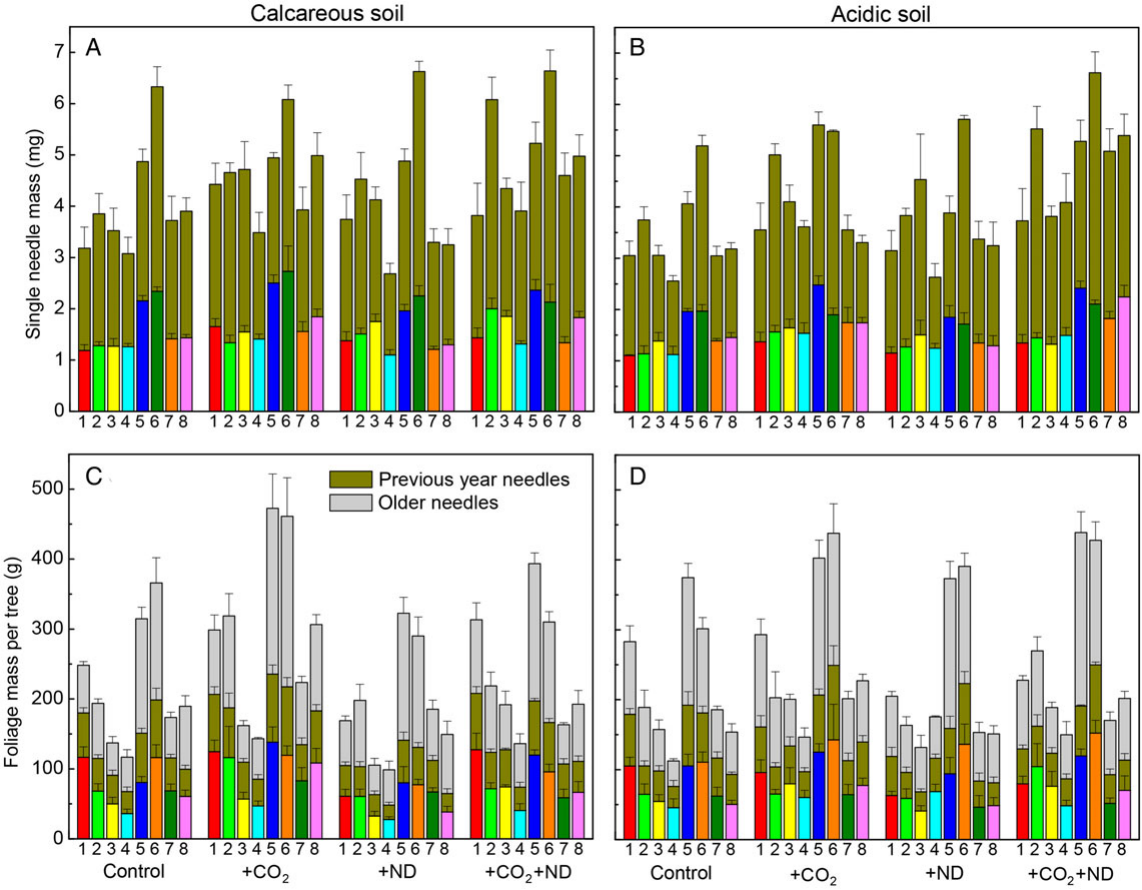
The change in the dry mass of single needles (A and B) and total crown foliage (C and D) in European spruce from eight origins growing together on either acidic or calcareous forest soil in response to +CO_2_, +ND and +CO_2_ + ND versus control (mean values + SE, *N* = 4, September harvest). Origins: 1 Harzvorland (red column), 2 Hochsauerland (green column), 3 Frankenwald (yellow), 4 Carpathia (cyan), 5 Kerns (blue), 6 Neuwilen (dark green), 7 Bremgarten (orange), 8 Maschwanden (magenta).

The +CO_2_ treatment strongly modified the morphology of spruce needles, whereas modifications by +ND and soil type were small and mostly not significant. In response to the +CO_2_ versus control treatment, the dry mass of current- and previous-year needles was increased by 25 and 23 % on average, respectively, and the needle area, thickness and LMA were increased by 14 % each (Fig. 5A and B, Table 2). The +CO_2_ treatment also lowered the water content of current-year needles, especially on the acidic soil (−5 %), whereas previous-year needles, water content of which was 7 % lower than in current foliage, showed no change. By the end of the experiment, the +CO_2_ treatment increased the total foliage biomass at tree level on average by +24 %, whatever the needle age (Fig. 5C and D, Table 2), varying for individual tree origins from 9 to 44 % without an apparent role of the plant material ontology or age.

**Table 2.** Analysis of variance, significance levels of factors affecting spruce foliage parameters. Effects (*F*-values above with df as subscripts, *P*-values, significant in bold, in parentheses, ↑ increasing, ↓ decreasing) of treatments (+CO_2_, +ND), season (harvest month September versus July), plant origin and soil type (acidic versus calcareous), number of repetitions each = 4, on the crown total, current and older foliage per tree or single needle biomass, morphology, biochemistry and needle element concentration. LMA, leaf dry mass per area; OPC, oligo-proanthocyanidins; PPC, polymerized proanthocyanidins. The interaction CO_2_ × ND was not significant in any case.

		Season	Origin	+CO_2_	+ND	Soil	Treatment × soil interactions
Tree	Total foliage	–	53.9_7,167_ (**<0.001**)	45.1_1,167_ (**<0.001**)↑	8.7_1,167_ (**0.025**)↑	0.4_1,167_ 0.566	
	Current-year foliage	–	21.2_7,167_ (**<0.001**)	20.0_1,167_ (**0.004**)↑	5.9_1,167_ 0.051	0.7_1,167_ 0.425	
	Previous-year foliage	–	26.6_7,167_ (**<0.001**)	14.0_1,167_ (**0.010**)↑	8.5_1,167_ (**0.027**)↑	0.0_1,167_ 0.974	
	Older foliage	–	60.4_7,167_ (**<0.001**)	17.0_1,167_ (**0.006**)↑	0.3_1,167_ 0.590	0.1_1,167_ 0.745	
Single needle current year	Mass	0.1_1,359_ 0.773	137.1_7,359_ (**<0.001**)	99.9_1,359_ (**<0.001**)↑	0.0_1,359_ 0.843	0.3_1,359_ 0.628	
	Area	1.7_1,71_ 0.201	14.6_1,71_ (**<0.001**)	6.7_1,71_ (**0.041**)↑	1.0_1,71_ 0.358	2.9_1,71_ 0.115	
	Thickness	0.1_1,71_ 0.832	10.8_1,71_ (**0.002**)	7.1_1,71_ (**0.038**)↑	0.1_1,71_ 0.836	0.0_1,71_ 0.926	
	% water	9.5_1,359_ (**0.002**)↓	6.7_7,359_ (**<0.001**)	16.4_1,359_ (**0.007**)↓	0.1_1,359_ 0.777	28.0_1,359_ (**<0.001**)↓	ND × soil 11.6_1,359_ (**0.005**)
	LMA	0.6_1,71_ 0.434	3.0_1,71_ 0.090	15.5_1,71_ (**0.008**)↑	2.2_1,71_ 0.186	8.9_1,71_ (**0.011**)↑	
	Colour	214.8_1,359_ (**<0.001**)↑	10.2_7,359_ (**<0.001**)	23.2_1,359_ (**0.003**)↓	21.6_1,359_ (**0.004**)↑	168.3_1,359_ (**<0.001**)↓	ND × soil 13.2_1,359_ (**0.003**)
	Chlorophyll *a* + *b*	–	0.5_1,48_ 0.491	5.8_1,48_ (**0.020**)↓	0.9_1,48_ 0.354	28.6_1,48_ (**<0.001**)↓	
	α + β carotenoids	–	0.1_1,48_ 0.709	6.2_1,48_ (**0.016**)↓	0.2_1,48_ 0.650	41.6_1,48_ (**<0.001**)↓	
	OPC	5.1_1,40_ (**0.029**)↓	–	9.2_1,40_ (**0.004**)↑	0.3_1,40_ 0.565	2.0_1,40_ 0.161	
	PPC	1.7_1,40_ 0.203	–	6.9_1,40_ (**0.012**)↑	0.7_1,40_ 0.421	2.1_1,40_ 0.271	
	PPC cell wall	1.8_1,40_ 0.187	–	1.1_1,40_ 0.311	1.3_1,40_ 0.271	4.8_1,40_ (**0.035**)↓	
Element	C	0.3_1,119_ 0.603	2.5_3,119_ 0.066	0.5_1,119_ 0.511	0.8_1,119_ 0.402	1.7_1,119_ 0.214	
	N	11.2_1,119_ (**<0.001**)↑	13.9_3,119_ (**<0.001**)	108.6_1,119_ (**<0.001**)↓	67.1_1,119_ (**<0.001**)↑	249.9_1,119_ (**<0.001**)↓	
	C/N	85.1_1,119_ (**<0.001**)↓	16.0_3,119_ (**<0.001**)	189.1_1,119_ (**<0.001**)↑	110.8_1,119_ (**<0.001**)↓	297.1_1,119_ (**<0.001**)↑	CO_2_ × soil 12.6_1,119_ (**0.004**)
	Ca	317.3_1,119_ (**<0.001**)↑	46.3_3,119_ (**<0.001**)	1.0_1,119_ 0.361	1.1_1,119_ 0.338	84.5_1,119_ (**<0.001**)↓	
	Fe	25.6_1,119_ (**<0.001**)↓	4.4_3,119_ (**0.006**)	0.8_1,119_ 0.402	0.1_1,119_ 0.824	0.2_1,119_ 0.669	ND × soil 27.1_1,119_ (**<0.001**)
	K	80.0_1,119_ (**<0.001**)↑	6.8_3,119_ (**<0.001**)	5.3_1,119_ 0.061	33.6_1,119_ (**0.001**)↓	14.8_1,119_ (**0.002**)↓	
	Mg	93.7_1,119_ (**<0.001**)↑	35.3_3,119_ (**<0.001**)	7.8_1,119_ (**0.031**)↓	1.5_1,119_ 0.271	33.0_1,119_ (**<0.001**)↑	CO_2_ × soil 6.1_1,119_ (**0.028**)
	Mn	141.6_1,119_ (**<0.001**)↑	67.7_3,119_ (**<0.001**)	0.3_1,119_ 0.629	5.9_1,119_ (**0.050**)↓	808.2_1,119_ (**<0.001**)↑	ND × soil 14.8_1,119_ (**0.002**)
	P	6.9_1,119_ (**0.001**)↑	1.9_3,119_ 0.128	2.5_1,119_ 0.165	147.6_1,119_ (**<0.001**)↓	126.1_1,119_ (**<0.001**)↓	CO_2_ × soil 17.7_1,119_ (**0.001**)
	P/N	0.2_1,119_ 0.627	1.1_3,119_ 0.357	0.4_1,119_ 0.554	11.0_1,119_ (**0.016**)↓	0.0_1,119_ 0.952	
	S	170.4_1,119_ (**<0.001**)↑	11.2_3,119_ (**<0.001**)	67.7_1,119_ (**<0.001**)↓	12.2_1,119_ (**0.013**)↓	48.8_1,119_ (**<0.001**)↓	
	Zn	292.7_1,119_ (**<0.001**)↑	42.8_3,119_ (**<0.001**)	23.4_1,119_ (**0.003**)↑	260.7_1,119_ (**0.002**)↓	55.2_1,119_ (**<0.001**)↑	
Single needle previous year	Mass	–	61.6_7,359_ (**<0.001**)	59.5_1,359_ (**<0.001**)↑	24.2_1,359_ (**0.003**)↑	3.6_1,359_ 0.080	
	Area	–	25.1_7,167_ (**<0.001**)	91.4_1,167_ (**<0.001**)↑	59.1_1,167_ (**<0.001**)↑	3.7_1,167_ 0.076	
	Thickness	–	2.2_1,31_ 0.151	43.8_1,31_ (**<0.001**)↑	1.2_1,31_ 0.314	0.13_1,31_ 0.720	
	% water	–	4.5_7,359_ (**<0.001**)	0.2_1,359_ 0.652	0.1_1,359_ 0.827	6.5_1,359_ (**0.024**)↓	
	LMA	–	21.4_7,167_ (**<0.001**)	27.6_1,167_ (**0.002**)↑	2.5_1,167_ 0.168	3.0_1,167_ 0.106	
	Colour	22.4_1,358_ (**<0.001**)↑	13.3_7,358_ (**<0.001**)	1.8_1,358_ 0.231	2.0_1,358_ 0.207	123.3_1,358_ (**<0.001**)↓

**Figure 6.**
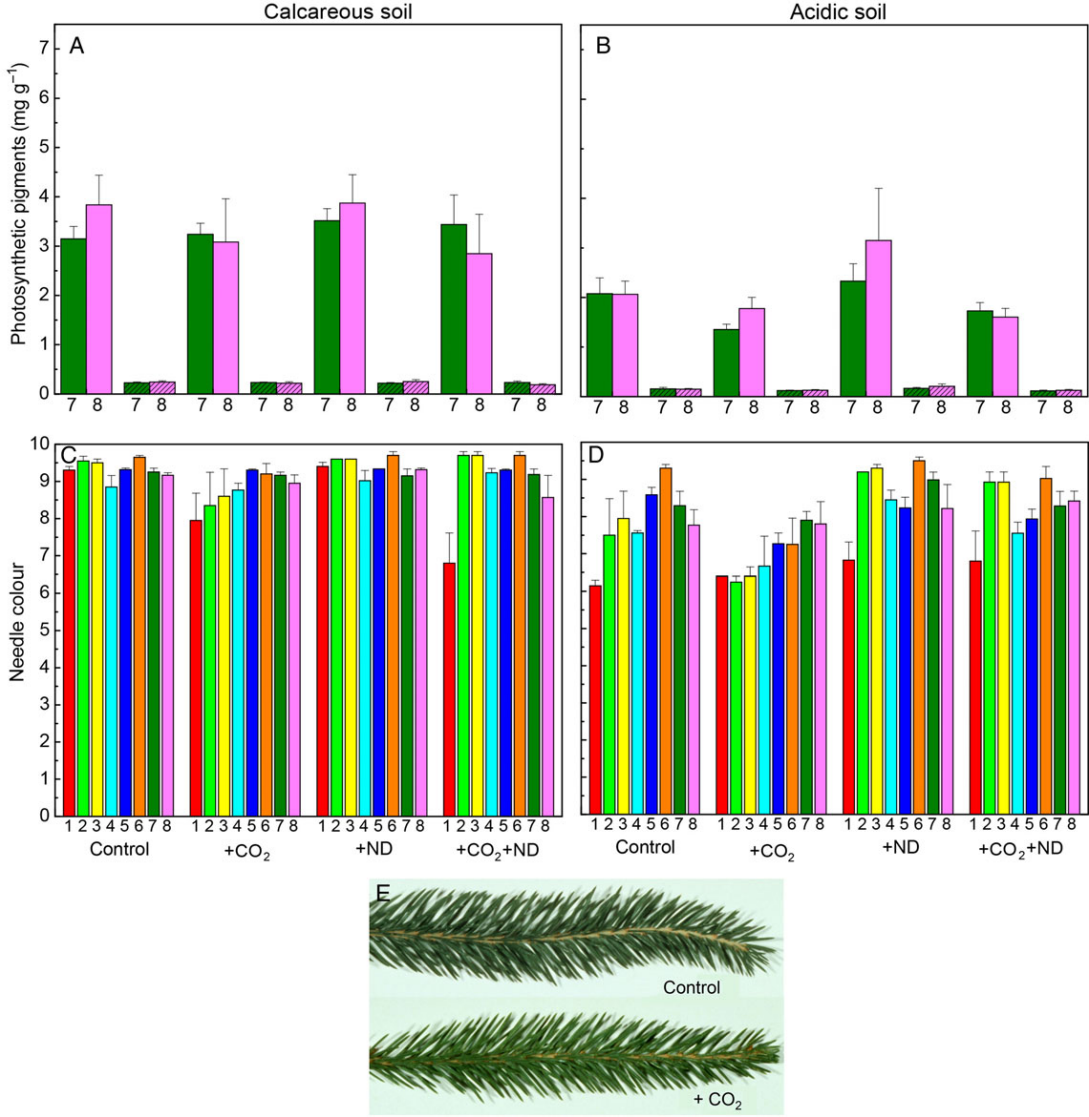
The change in the concentration of photosynthetic pigments in July (A and B), namely chlorophyll *a* + *b* and α + β carotenoids (hatched columns), and in the needle colour in September (C and D) in the current-year foliage of several European spruce origins (bar colours = Fig. 5) growing on either acidic or calcareous forest soil in response to +CO_2_, +ND and +CO_2_ + ND versus control (mean values + SE, *N* = 4). (E) Typical examples of needle discolouration in response to elevated CO_2_.

**Figure 7.**
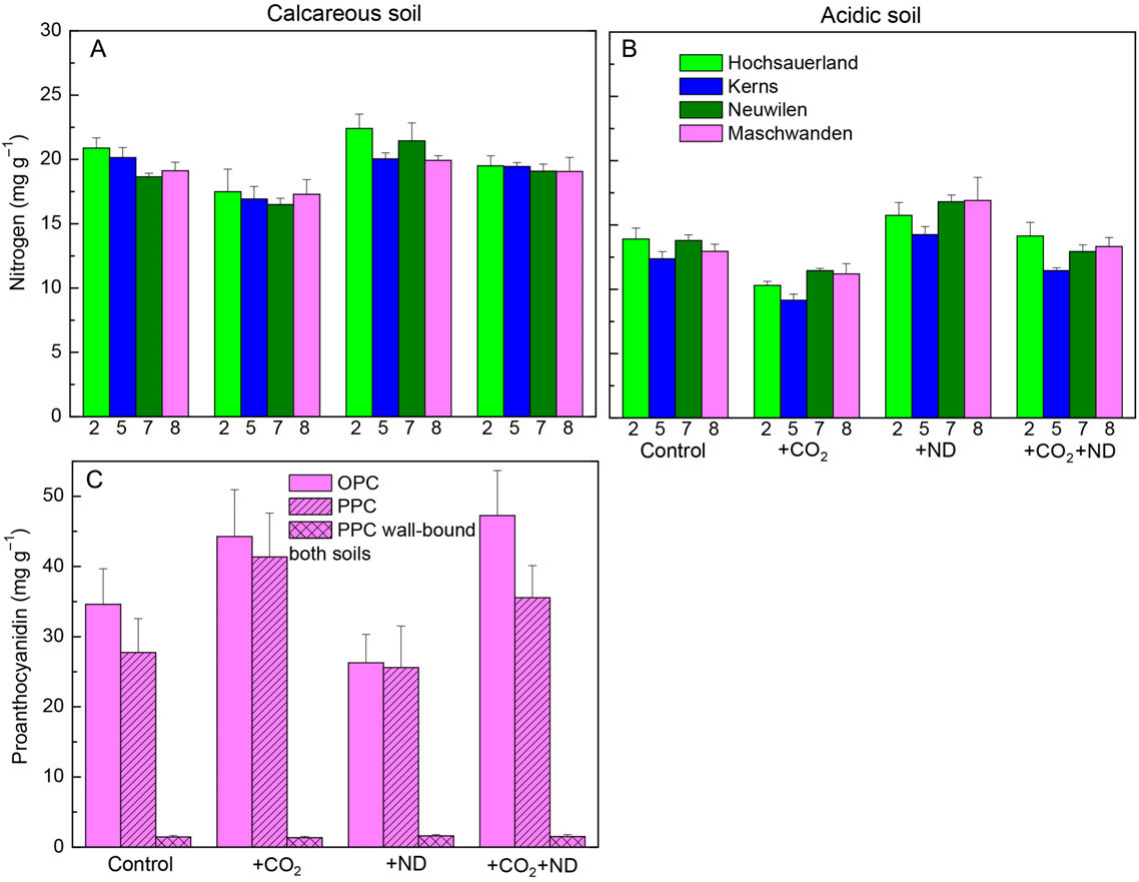
Change in the concentration of nitrogen (A and B) and PC (C) oligomers (OPC) and polymers (PPC) in September in response to +CO_2_, +ND and +CO_2_ + ND versus control, within the foliage of several spruce origins (bar colours = Fig. 5, C: origin number 8 only) growing on either acidic or calcareous forest soil (C: soil types without effect, data pooled) (mean values + SE, A and B: *N* = 4, C: *N* = 8).

The exposure to +CO_2_ reduced the pigment content and leaf colour of spruce needles, with a variation among the origins. The concentration of photosynthetic pigments within current-year needles in July was decreased by +CO_2_ (chlorophyll, 21 %; carotenoids, 18 %), whereas the +ND treatment caused no significant change (Fig. 6A and B, Table 2). The soil had an important influence. The concentration of chlorophyll and carotenoids on the acidic versus calcareous soil was by 40 and 36 % lower, respectively. The current-year foliage under +CO_2_ showed a lighter green colour (Fig. 6A, B and E) and this discolouration was increased on the acidic versus calcareous soil (−7 and −4 %) whereas the +ND led to darker green hue on acidic soil (+11 and + 2 %, significant ND × soil interaction; Fig. 6C and D, Table 2). Belying this interaction in the needle colour, the N concentration of current-year needles was significantly decreased by +CO_2_ (−15 %, nearing, on the acidic soil, the deficiency level of <11.8 mg g^−1^, according to Mellert and Göttlein 2012) but increased by +ND (+15 %, Fig. 7A and B). Needle colour evolved during the vegetation season and samples harvested in September versus July showed darker green hues (current-year needles +13 %, previous year +7 %, Table 2).

The concentration of P was decreased in the needles by ND (−17 %), resulting in a decreased P/N ratio (−23 %, Table 2), but remained, together with all other elements showing minor changes in response to treatments, within the normal range reported by Mellert and Göttlein (2012). On the acidic versus calcareous soil, the needle concentration of Mn was multiplied 26 times, the C/N ratio, Mg and Zn concentration were by 42/15 and 44 % higher and the concentration of Ca/N/P by 33/28/24 % lower. In September versus July, the concentration of Ca/Mn/Zn was by 72/90/88 % higher.

**Figure 8.**
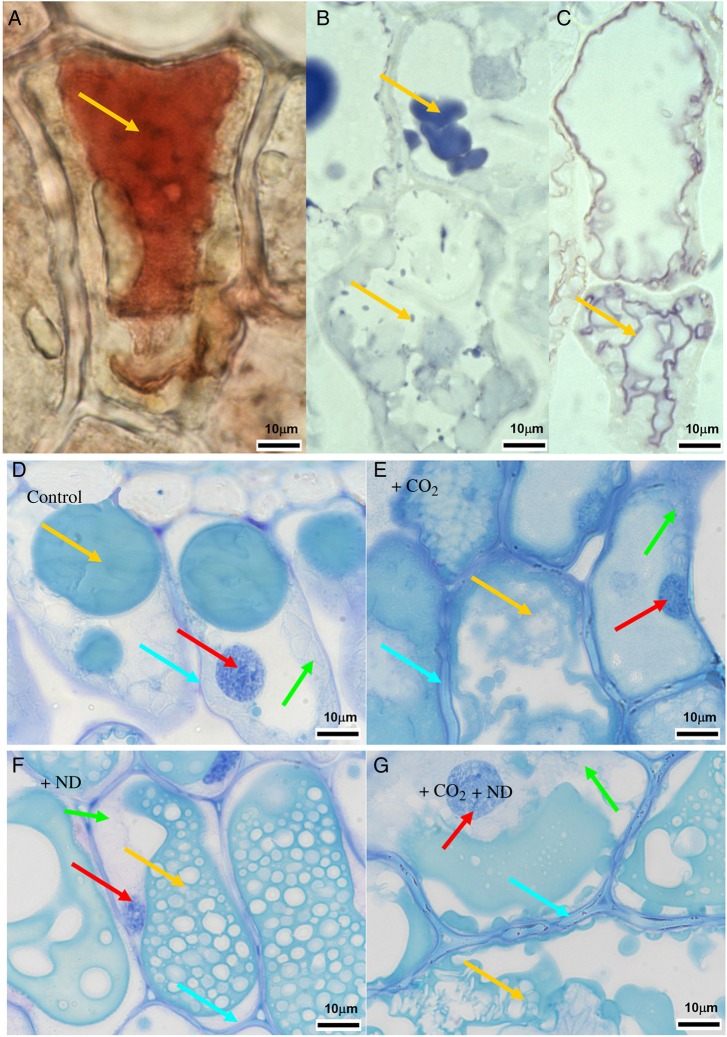
Histochemical detection of PCs in mesophyll cells of current-year spruce needles using vanillin (A) and DMACA (B and C) reagents. Procanthocyanidin oligomers (staining red in A) were mostly soluble in the vacuolar medium. PC polymers (staining bluish in B and C) were precipitated as phenolic bodies of varying size and shape such as globules (B) or fine ribbons bordering the tonoplast (C). Structural effects of +CO_2_ (E), +ND (F) and +CO_2_ + ND (G) versus control (D) in mesophyll cells from samples harvested in January (staining with toluidine blue). There were no starch grains in chloroplasts (green arrows), whatever the treatment, since this relates to a typical winter dormancy effect. Under +CO_2_, cell walls (cyan arrows) were thickened (E, G versus D), the frequency of chloroplasts (green arrows) reduced (E, G versus D, F) and the amounts of condensed tannins (yellow arrows) increased (E, G versus D), with most of cell lumen filled up. Specific to the +CO_2_ treatment (E), vacuoles were enlarged and had an irregular shape whereas the cytoplasm and nucleus (red arrows) showed increased condensation. In response to +ND and as a tendency, nuclear chromatin appeared condensed and the amounts of condensed tannin increased (F versus D).

The PC concentration in needles and the needle structure responded independent of soil type, but primarily only to the +CO_2_ treatment. The concentration of soluble OPC and PPC was sizeably increased by +CO_2_ (+55 and +48 %), whereas +ND had no effect (Fig. 7C, Table 2). The insoluble PPC (cell wall-bound PPC), the amounts of which in September and July reached 4.3 and 4.6 %, respectively, of those of soluble PPC, were not changed by the treatments. Histochemically, the OPC and PPC showed distinct structural traits irrespective of the harvest in July and September. A typical appearance of PC still prevailing in January is shown in Fig. 8D–G, when the cell structure showed typical dormant traits as indicated by the missing starch grains. The OPC were soluble in the vacuole medium, as indicated by homogeneous organelle filling (Fig. 8A) whereas the PPC were segregated in the form of globular (Fig. 8B), ribbon-like (Fig. 8C) or sponge-like (Fig. 8F) solid bodies. Whatever the soil type, tree origin and harvest date, the +CO_2_ treatment increased apparently the PC amounts within vacuoles of mesophyll cells (Fig. 8E versus D). Other changes within needle mesophyll in response to +CO_2_ included the enlargement and irregular periphery of vacuoles, condensation of cytoplasm and nucleus, thickening of cell walls and a tendency to increased intercellular accumulation of Ca-oxalate crystals (Fig. 8E versus D). These structural changes were indicative of moderate degenerative processes. In response to +ND, only an increased PC accumulation and a tendency to more nucleus condensation was observed (Fig 8F versus D) whereas the effects of +CO_2_ + ND were similar to those of +CO_2_ alone (Fig. 8E and G).

There was a large, highly significant difference between spruce and beech in all parameters except OPC and PPC (Table 3). Beech and spruce showed contrasting reactions to the treatments with significant interactions: species × CO_2_ for total foliage mass, single leaf mass, thickness, water concentration and C/N ratio and species × ND for single leaf mass, LMA, C/N, P/N, K, Mg, S and Zn concentrations (Table 3). The interaction species × soil was always significant except for leaf thickness, PPC and P/N, that of species × season was significant except for water, LMA, C, K, Mn and P (Table 3). Only responses of foliar P, pigments PC and N concentrations showed parallel response to +CO_2_ and +ND in both species. Only spruce profited from an elevated supply of C with a remarkable increase in the total foliage mass, together with that of single needles and irrespective of soil type. Beech, however, showed no significant change at the tree crown level and a lesser increase in individual leaf biomass with significant differences between both soil types. In response to +CO_2_, the foliar organs of both species showed consistent trends of discolouration, although needle colour evolved during the season in contrast to beech leaves. Similarly, leaf pigment concentrations and N concentration decreased and were, depending on species and parameter, partly compensated by +ND. The concentration of vacuolar OPC and PPC fraction within foliage of both species was similar, whereas the cell walls of beech leaves contained 1.4 times more PPC than those of spruce. The PC of the cell walls, as indicated by increased LMA and observed microscopically, formed important carbon sinks in foliage of both species for supplementary assimilates in response to elevated carbon availability. Within both species, changes in the foliar concentration of nutrients were similar. The spruce needle versus beech leaf showed smaller differences between soil types but higher accumulative levels of Ca/Mn/Zn, the foliar concentration of which was increased during the vegetation season. At cell level, both species showed similar reactions to the +CO_2_ treatment including cell wall thickening and degenerative changes within mesophyll cells, irrespective of soil type and tree origin.

**Table 3.** Analysis of variance, significance levels of the species difference in crown foliage per tree, single leaf parameters, leaf pigments, leaf PPs and leaf elemental concentrations. *F*-values and *P*-values (significant in bold) for species and their interactions with the treatments, soil type and season (July, September harvest). Abbreviations as in Tables 1 and 2.

	Variable	df	Species		Species × CO_2_		Species × ND		Species × soil		Species × season	
			*F*	*P*	*F*	*P*	*F*	*P*	*F*	*P*	*F*	*P*
Tree	Total foliage	1,56	1145.9	**<0**.**001**	28.1	**<0**.**001**	1.6	0.206	40.3	**<0**.**001**	–	
Single leaf	Mass	1,118	7367.3	**<0**.**001**	9.0	**0**.**003**	5.0	**0**.**028**	73.2	**<0**.**001**	7.4	**0**.**007**
	Area	1,118	11188.2	**<0**.**001**	2.1	0.153	1.9	0.177	34.8	**<0**.**001**	10.1	**0**.**002**
	Thickness	1,118	2744.0	**<0**.**001**	19.2	**<0**.**001**	0.4	0.526	0.7	0.409	25.7	**<0**.**001**
	% water	1,118	1422.7	**<0**.**001**	12.4	**<0**.**001**	0.3	0.586	17.9	**<0**.**001**	2.3	0.134
	LMA	1,118	533.9	**<0**.**001**	3.8	0.055	6.8	**0**.**011**	49.8	**<0**.**001**	0.0	0.906
	Colour	1,118	78.1	**<0**.**001**	0.5	0.471	0.3	0.588	31.1	**<0**.**001**	79.3	**<0**.**001**
	Chlorophyll *a*+*b*	1,55	39.2	**<0**.**001**	0.0	1.0	1.8	0.187	5.8	**0**.**020**	–	
	α+β carotenoids	1,55	6.5	**0**.**014**	0.0	1.0	0.9	0.355	17.0	**<0**.**001**	–	
	OPC	1,97	3.9	0.051	1.2	0.283	3.1	0.081	8.5	**0**.**004**	6.6	**0**.**012**
	PPC	1,97	2.7	0.101	3.2	0.078	1.6	0.205	1.8	0.182	10.1	**0**.**002**
	PPC cell wall	1,97	10.2	**0**.**002**	0.5	0.481	0.7	0.410	0.7	0.404	17.7	**<0**.**001**
Element	C	1,118	19.5	**<0**.**001**	0.0	0.973	0.7	0.308	16.5	**<0**.**001**	2.8	0.097
	N	1,118	787.4	**<0**.**001**	0.9	0.349	1.0	0.319	24.6	**<0**.**001**	65.8	**<0**.**001**
	C/N	1,118	777.2	**<0**.**001**	24.6	**<0**.**001**	13.8	**<0**.**001**	114.4	**<0**.**001**	44.0	**<0**.**001**
	Ca	1,118	1151.1	**<0**.**001**	0.1	0.808	1.5	0.221	129.4	**<0**.**001**	43.9	**<0**.**001**
	Fe	1,118	70.6	**<0**.**001**	0.0	0.883	0.3	0.609	4.8	**0**.**030**	48.6	**<0**.**001**
	K	1,118	11.6	**<0**.**001**	1.0	0.328	9.7	**0**.**002**	33.58	**<0**.**001**	0.3	0.601
	Mg	1,118	1136.7	**<0**.**001**	3.0	0.085	5.4	**0**.**021**	265.5	**<0**.**001**	42.6	**<0**.**001**
	Mn	1,118	385.0	**<0**.**001**	0.1	0.779	2.2	0.140	374.2	**<0**.**001**	0.3	0.584
	P	1,118	79.0	**<0**.**001**	3.8	0.053	2.9	0.090	49.8	**<0**.**001**	0.8	0.371
	P/N	1,118	164.7	**<0**.**001**	0.2	0.692	4.3	**0**.**041**	0.9	0.482	3.9	0.051
	S	1,118	1041.6	**<0**.**001**	1.6	0.210	13.6	**<0**.**001**	7.7	**0**.**006**	83.5	**<0**.**001**
	Zn	1,118	71.8	**<0**.**001**	2.3	0.134	11.6	**<0**.**001**	17.8	**<0**.**001**	57.3	**<0**.**001**

## Discussion

### Fertilization effect by elevated CO_2_ and the constraint of nutrient availability

During the 4 experimental years, the elevated CO_2_ concentrations acted as a fertilizer within the foliage of treated trees, but the effects varied, primarily, as a function of the species with significant modifications by the tree origins. Thus, these findings confirmed, on an experimental basis, the response plasticity of different species and origins of forest trees to elevated CO_2_. With the two species growing competitively, spruce showed a consistently positive response to +CO_2_ at the needle and tree crown level whereas for beech only leaf size showed a small increase on calcareous soil. Hence, these findings are in line with models predicting a superior growth increase for coniferous versus deciduous trees within a CO_2_-richer environment (Tatarinov *et al*. 2011). Mechanistically within foliage, the discrepancy between the results for long-living spruce needles and deciduous beech leaves may relate to the increased water-use efficiency of conifer needles because of their more compact cell structure which limits internal CO_2_ diffusion (Niinemets *et al*. 2011). The increased water-use efficiency, as a consequence of exposure to elevated CO_2_, was measured in ICAT also at the ecosystem level (Sonnleitner *et al*. 2001) and has been found in other experimental studies (Battipaglia *et al*. 2013). However, soil type and especially nutrient availability also contributed to further differentiating the responses between beech and spruce and, primarily in beech, mediating the CO_2_ fertilization effect. The importance of nutrient availability including the soil type for carbon fertilization is in agreement with previous findings at the model ecosystem level from the present experiment (Hagedorn *et al*. 2002) and from other experimental studies (Norby and Zak 2011), and is also indicated by modelling (De Vries and Posch 2011). The weaker CO_2_ fertilization effect in beech was related to the insufficient supply of the key nutrient element N, particularly on acidic soil. But, because foliage mass per tree or leaf/needle size was not correlated to foliar N concentration, the hypothesis that N was diluted by an enhanced growth due to +CO_2_ (Eller *et al*. 2011) has to be refuted at the foliage level. The same was recently reported for different ecosystems (Feng *et al*. 2015). In contrast to spruce, beech responded positively to the +ND treatment which, together with +CO_2_, showed significant interactions with the soil type at the crown and single leaf levels. Furthermore, changes in the P/N ratios and other essential nutrients (S, Mg) might partly explain why cell wall polysaccharides and PC compounds, less demanding regarding the nutrient supply, formed important sinks for the supplementary fixed carbon*.* Consequently, within a CO_2_-richer atmosphere in the future, the carbon storage capacity of forest trees may be reduced. The findings here, however, suggest that important differences between species, representing coniferous and deciduous trees, should be expected as a consequence of varying tolerance to changes in the nutrient supply. However, no element dropped below the concentration deficiency limit and leaf discolouration symptoms, with respect to those to be found in the case of nutrient deficiency, missed specificity (Vollenweider and Günthardt-Goerg 2006; Hartmann *et al*. 2007). Confronted with the same soil conditions, the better responsiveness of spruce versus beech to the +CO_2_ treatment and its relative insensitivity to changes in nutrient ratios and N fertilization could relate to its long-lived foliage and wider tolerance regarding changes in the nutrient supply and broader ecological niche (Härdtle *et al*. 2004). Interestingly, a trend towards decreasing foliar P concentrations in European forests has been recently related to enhanced CO_2_ and N deposition (Jonard *et al*. 2015), whereas in the present experiment the concentration of P was only decreased by +ND, but not changed by +CO_2_ in both species.

As trees age, experiments in mature stands suggest that the fertilization gain evidenced in the present study could fade as a consequence of intra- and interspecific competition and decreasing tree sensitivity (Bader *et al*. 2013). Furthermore, long-term effects on soil properties in response to elevated CO_2_, e.g. a pH increase (Rennenberg *et al*. 2010), may further constrain the response of forest trees.

### Stress reactions in foliage because of nutrient imbalance

In response to the +CO_2_ treatment, and as a likely consequence of nutrient imbalance, stress reactions were observed primarily within beech leaves but also within spruce needles, albeit with a lower intensity. The most prominent stress symptom was discolouration of foliar organs which was associated with decreased concentrations of photosynthetic pigments and foliar N. Such changes in the course of the vegetation season are indicative of ACS processes within foliage (Pell *et al*. 1999; Günthardt-Goerg and Vollenweider 2007). Discoloured foliage (Mousseau and Enoch 1989; Utriainen and Holopainen 1998; Ormrod *et al*. 1999; Cavender-Bares *et al*. 2000; Sallas *et al*. 2003; Hirano *et al*. 2012) and a lowered N concentration (Hättenschwiler and Körner 1998; Cavender-Bares *et al*. 2000; Jach *et al*. 2000; Lindroth *et al*. 2001; Cao *et al*. 2008) have frequently been observed in response to elevated CO_2_. Regarding the other retrievable elements, which also have high phloem mobility, their concentration was decreased in response to +CO_2_ only for Mg and S whilst others (K, P) remained unaffected thus indicating that element retrieval because of ACS differed to that occurring during autumnal senescence (Eschrich *et al*. 1988; Marschner 1995). Similarities between ACS and the ageing process in foliage were further confirmed by the changes in elemental concentrations between September and July with a decrease in retrievable elements (N, S and Mg) in beech and an increase in those which low phloem mobility force to accumulate during the vegetation season in both species, namely Ca, Mn, Zn and Fe (in beech only).

Degenerative changes in the cell structure, stronger in beech than in spruce, were also indicative of stress reactions confirming the ACS diagnosis. Stress symptoms typical of ACS (Günthardt-Goerg *et al*. 1993; Vollenweider *et al*. 2003; Günthardt-Goerg and Vollenweider 2007) in response to +CO_2_ thus included the (i) decreased frequency of chloroplasts, (ii) degenerated chloroplasts as visualized in TEM imaging showing poorly resolved grana and thylacoid structures and the increased density of plastoglobuli, (iii) condensation of cytoplasm and nuclear material and (iv) vacuole enlargement and increased plastoglobuli segregation. However, the larger cell and starch grain size observed in beech in response to +CO_2_ and +CO_2_ + ND or the increased LMA did not form stress symptoms but they may represent, especially in the latter case, a characteristic response to the aforementioned treatments. Some structural markers of ACS have also been observed in other studies, after exposure of deciduous trees (Oksanen *et al*. 2005) or conifers (Utriainen and Holopainen 1998; Sallas *et al*. 2003) to elevated levels of CO_2_. Cell wall thickening, confirmed by increased LMA, has, to our knowledge, only been reported for birch (Oksanen *et al*. 2005). However, the late stages of senescence, which proceed according to a genetically controlled cell death programme (typical markers in mesophyll cells: reduction of grana size and frequency within chloroplast, heavy accumulation of plastoglobuli, increased vacuolation of cytoplasm, disruption of membranes and degradation of organelle content; Mikkelsen and Heide-Jørgensen 1996; Fink 1999; Orzaez and Granell 2004; Kivimäenpää and Sutinen 2007) were not present in our study. Similarly, no premature abscission of foliage was observed, but synergistic interactions between ACS processes and autumnal senescence cannot be totally excluded on the basis of the presented data.

### Changes in the concentration of secondary metabolites

The increased concentration of PC measured in response to +CO_2_ in both species (in beech also to +ND), and observed within vacuoles of mesophyll cells, formed an important indication of degenerative changes triggered by nutrient imbalances. Although the PC analyses from one tree origin may not be quantitatively representative for the species, the mechanisms in response to the treatment are. Indeed, PC are important defense compounds accumulated in response to various biotic and abiotic stress factors and they are frequently associated with ACS processes, although not being primarily a senescence marker (Seigler 1998; Fink 1999; Günthardt-Goerg and Vollenweider 2007). At the cell level, vacuolar accumulation of PC was found amid many other stress indicators within cells and they were thus part of ACS processes. In response to elevated CO_2_, higher concentrations of total phenols have been reported in at least 19 different plant species (Peñuelas *et al*. 1997; Tognetti and Johnson 1999; Sallas *et al*. 2003). With a biomass fraction amounting to 5.6 % (beech) and 6.1 % (spruce) out of a total phenol fraction of 12 and 6.9 %, respectively (results not shown), the PC represented the principal phenolic compound to be found in beech and spruce foliage. Hence, an increase in these already high amounts of secondary metabolites probably enhanced the defensive capacities in beech and spruce foliage, as often observed within constraining environments (Fink 1999).

Similar to LMA, another function of enhanced PC synthesis could be to provide a sink for supplementary-fixed carbon whilst limited N stocks and other nutrients limited more demanding metabolic pathways. Indeed and according to the partially overlapping theories trying to explain, for example, how environmental CO_2_ variations can affect the concentration of secondary metabolites (Jones and Hartley 1999; Bezemer *et al*. 2000; Mattson *et al*. 2005; Matyssek *et al*. 2012), the higher PC concentration in response to treatments could result from (i) an increased differentiation period, the treatments being perceived as at least partly unfavourable change in the environmental conditions (GDBH); (ii) an imbalance between the C and N supply (carbon-nutrient balance hypothesis, CNBH) and/or (iii) a lower protein demand because of slowed-down growth and increased availability of phenylalanine substrate (protein competition model, PCM). In the case of beech, given the observed responsiveness to N fertilization and stress symptoms, CNBH > GDBH ≥ PCM appear to best explain the observed PC increase. Given its superior tolerance to changes in the nutrient supply, lesser response to the +ND treatment and moderate stress reactions, the interpretation for spruce is slightly different with GDBH ≥ CNBH > PCM. Also in the case of the mechanism driving the accumulation of secondary metabolites, beech and spruce showed characteristic specificities.

## Conclusions

Exposure of young deciduous beech and coniferous spruce trees to elevated levels of CO_2_ during the 4 years of experimentation had a rather positive fertilizing effect within the foliage but with significant differences between both species. This fertilization effect was partly mediated by the soil type and nitrogen supply, and weakened as a consequence of nutrient imbalance leading to stress reactions, still poorly studied. Differences between the two tree species regarding the latter soil mediation effect, responsiveness to N fertilization and stress reactions were in agreement with our first hypothesis (i.e. CO_2_ effect vary according to species, nitrogen supply and soil type) which was thus validated. Hypothesis 2 (i.e. changed nutrient demand because of an enhanced CO_2_ supply remediated by elevated ND) was confirmed by findings in beech but should be rejected regarding spruce. Finally, a decrease in fertilization gain because of ACS reactions in foliage of both species (hypothesis 3), stronger in beech, can partly explain the lower responsiveness of beech versus spruce to an elevated CO_2_ supply. Hence, findings shed light on mechanisms, namely an acceleration of cell senescence in foliage and changed carbon sink at a cellular level threatening the potential increase of carbon fixation in the foliage of trees within a CO_2_-richer environment in the future. Contrasting growth reactions between deciduous and evergreen species to climate change but similar reactions at the leaf cell level are to be expected.

## Sources of Funding

This experiment was a part of the Swiss contribution to COST 614 for which financial support by the Swiss Federal Office for Education and Science is gratefully acknowledged.

## Contributions by the Authors

Both authors have equally contributed to the manuscript and research presented.

## Conflict of Interest Statement

None declared.
